# Comparable outcomes among trial and nontrial participants in a clinical trial of antibiotics for childhood pneumonia: a retrospective cohort study

**DOI:** 10.1016/j.jclinepi.2017.10.016

**Published:** 2018-02

**Authors:** Ambrose Agweyu, Jacquie Oliwa, David Gathara, Naomi Muinga, Elizabeth Allen, Richard J. Lilford, Mike English

**Affiliations:** a Department of Public Health Research, Health Services Unit, KEMRI-Wellcome Trust Research Programme, P.O. Box 43640, 00100 Nairobi, Kenya; b Department of Medical Statistics, London School of Hygiene and Tropical Medicine, Keppel Street, London WC1E 7HT, UK; c Department of Health Sciences, Warwick Medical School, University of Warwick, Medical School Building, Coventry CV4 7AL, UK; d Nuffield Department of Medicine, University of Oxford, Old Road Campus, Headington, Oxford OX3 7BN, UK

**Keywords:** Acute respiratory infections, World Health Organization, Generalizability, Clinical trial, Amoxicillin, Benzyl penicillin, External validity

## Abstract

**Objectives:**

We compared characteristics and outcomes of children enrolled in a randomized controlled trial (RCT) comparing oral amoxicillin and benzyl penicillin for the treatment of chest indrawing pneumonia vs. children who received routine care to determine the external validity of the trial results.

**Study Design and Setting:**

A retrospective cohort study was conducted among children aged 2-59 months admitted in six Kenyan hospitals. Data for nontrial participants were extracted from inpatient records upon conclusion of the RCT. Mortality among trial vs. nontrial participants was compared in multivariate models.

**Results:**

A total of 1,709 children were included, of whom 527 were enrolled in the RCT and 1,182 received routine care. History of a wheeze was more common among trial participants (35.4% vs. 11.2%; *P* < 0.01), while dehydration was more common among nontrial participants (8.6% vs. 5.9%; *P* = 0.05). Other patient characteristics were balanced between the two groups. Among those with available outcome data, 14/1,140 (1.2%) nontrial participants died compared to 4/527 (0.8%) enrolled in the trial (adjusted odds ratio, 0.7; 95% confidence interval: 0.2–2.1).

**Conclusion:**

Patient characteristics were similar, and mortality was low among trial and nontrial participants. These findings support the revised World Health Organization treatment recommendations for chest indrawing pneumonia.

**What is new?**

**Key findings**
• In this study of hospitalized children with chest indrawing pneumonia, routinely collected observational data from nontrial patients provide estimates of clinical characteristics and mortality comparable to those of trial participants.

**What this adds to what was known?**
• Effectiveness of oral amoxicillin for the treatment of chest indrawing pneumonia has been demonstrated in clinical trials.
• The results of this analysis suggest generalizability of clinical trial data to routine settings where pneumonia mortality is perceived to be high.

**What is the implication and what should change now?**
• The findings lend support to the WHO revised recommendations and provide useful evidence for national programs implementing pneumonia case management in sub-Saharan Africa where uptake of the WHO guidelines has been poor.

## 1 Introduction

In the hierarchy of sources of evidence for guideline development, randomized controlled trials (RCTs) are generally regarded to be of high quality [1]. Critics of traditional RCTs, however, argue that the strict enrollment criteria and enhanced care provided to patients enrolled in clinical trials limit the external validity of the findings to routine clinical settings [2]. A “trial effect” demonstrating better overall outcomes among patients recruited in clinical trials, independent of the effectiveness of the intervention treatment, has previously been described [3]. This effect is arguably more pronounced in resource limited settings where routine care is often considerably poorer than care provided to participants of clinical trials assigned to receive “standard care.” In a large pragmatic trial on fluid resuscitation among febrile children in sub-Saharan Africa, mortality among control subjects was noted to have been 50% lower than that observed in routine practice before the trial [4].

For childhood pneumonia, the leading single cause of childhood mortality, data from large clinical trials conducted in predominantly Asian populations were used to inform a major revision of the World Health Organization (WHO) recommendations for empirical therapy. In the revised recommendations, children with indrawing pneumonia (lower chest indrawing in the absence of danger signs suggestive of more severe forms of disease) are to be managed as outpatients with oral amoxicillin in place of the previous guidelines requiring inpatient therapy with injectable penicillin [5]. Uptake of this policy has been poor across sub-Saharan Africa partly due to fears of the safety of home management of a population of children with a potentially high risk of mortality [6]. These concerns are partially addressed in a pragmatic noninferiority RCT of oral amoxicillin vs. benzyl penicillin conducted at six Kenyan hospitals [7]. In a parallel exercise, we collected observational data from patients who were treated for pneumonia at the participating hospitals while the trial was recruiting, but who did not take part in the trial. Reasons for not participating in the trial included refusal to provide consent or failure of the recruiting clinician to enroll the patient in the trial because they were admitted at night or during weekends when recruitment was suspended. Variations of this design have been previously applied in a limited number of studies conducted in high-income settings [8], [9].

We specifically sought to compare mortality among children enrolled in the RCT with a similar population of nontrial participants hospitalized at the same health facilities over the period the trial was conducted to address anticipated concerns from clinicians and policymakers regarding the generalizability of the trial results.

## 2 Methodology

### 2.1 Participants

We created a retrospective cohort of children admitted with chest indrawing pneumonia (WHO 2014 definition) at six Kenyan public hospitals. The primary independent variable was enrollment vs. nonenrollment in a RCT comparing the effectiveness of oral amoxicillin vs. benzyl penicillin for chest indrawing pneumonia [7]. The main outcome was mortality during the inpatient period. The study sites were average-sized hospitals with 2,500–4,500 pediatric admissions each year. Three of the facilities were located in malaria endemic regions of the country. Four of the sites were district-level hospitals, while two were provincial referral hospitals. All six hospitals were medical internship training centers with inpatient pediatric departments headed by at least one pediatrician.

### 2.2 Eligibility criteria

We included children aged between 2 and 59 months hospitalized with pneumonia at the study sites between September 2011 and August 2013. To overcome the potential effects of alternative antibiotic treatments on clinical outcome and the challenge of accurate identification of ineligible children with undocumented exclusion criteria, we restricted the study population to children with chest indrawing at admission who were treated with either benzyl penicillin alone at 50,000 IU/kg every 6 hours (observational study and control group of RCT) or oral amoxicillin at 40–45 mg/kg every 12 hours (intervention group of the RCT). Children with documented clinical diagnoses that would preclude the use of benzyl penicillin or amoxicillin monotherapy including severe acute malnutrition, HIV, meningitis, and WHO-defined very severe pneumonia were excluded. Caregivers who declined to provide written informed consent for enrollment were excluded from the clinical trial but were considered for inclusion in the observational cohort.

### 2.3 Sample size

A total of 527 children were enrolled in the clinical trial. Assuming a similar number of eligible participants were admitted at the study hospitals but not enrolled in the clinical trial, a sample size of 1,054 would provide 80% power to detect a twofold higher mortality among children who were not enrolled in the trial vs. those enrolled in the trial at a 5% significance level assuming an estimated mortality risk of 1% among children enrolled in the clinical trial based the results of a similar large multicountry clinical trial [10].

### 2.4 Study procedures

Clinical documentation for the observational cohort was not monitored as rigorously as it was in the clinical trial. However, considerable effort was made to ensure overall quality of clinical care and documentation through training for clinical staff on the pneumonia guidelines at the beginning of the study and at 3–6 monthly intervals, promotion of the use of structured patient admission forms during clinical assessment, and provision of the national pediatric guidelines to health workers. Staff also underwent a 5-day course designed for the dissemination of the Ministry of Health pediatric guidelines for health facilities called Emergency Triage Assessment and Treatment Plus [11] at various times during the study period.

Children participating in the RCT were enrolled prospectively by a trained study clinician after screening for eligibility including provision of written informed consent from caregivers. All trial participants were reviewed daily by the study clinician in consultation with a pediatrician based at the hospital. The trial protocol outlined specific criteria defining treatment failure among trial participants to which the clinical team were advised to adhere. The procedures specific to the clinical trial is described separately [7].

Data were entered daily in an online database that was reviewed by the trial principal investigator and study pediatrician for conflicting or missing observations which were communicated directly to the site clinician for prompt correction.

Trained research assistants extracted data for the nontrial participants retrospectively from hospital records. Supervision of the data abstraction exercise was provided onsite by the hospital pediatrician and a trained clinical officer with additional telephone and e-mail support from the study principal investigator.

The primary data collection tool was developed in REDCap, an open source web-based database application [12]. The structure of the database was based on the Ministry of Health's Pediatric Admission Record form [11] that captures data on patient sociodemographic and clinical characteristics, and admission diagnosis. Additional fields were included to record data on daily inpatient care including treatment prescribed and information on discharge diagnosis and clinical outcome (survival or death).

### 2.5 Statistical analysis

Data were inspected for distribution and completeness. Characteristics of the study population were summarized and compared by trial status (trial vs. nontrial participants). The univariate odds ratio for mortality between trial and nontrial participants was reported with an accompanying 95% confidence interval (CI). Potential causal pathways relating enrollment in the clinical trial, patient characteristics, and mortality were developed to determine variables that would be considered potential confounders. Variables that altered the strength of the primary association in bivariate analyses were selected for inclusion in the multivariate models along with age and hospital level which were regarded as a priori covariates. To account for missing data on variables selected for inclusion in the multivariable model, multiple imputation by chained equations was performed under a missing at random assumption. This method was favored for its flexibility in handling both categorical and continuous variables [13], [14]. To enhance efficiency of the imputation model, we included variables for which documentation was complete or almost complete.

## 3 Results

Medical records were available for 1,709 children aged 2–59 months hospitalized at the study sites between September 2011 and August 2013, with pneumonia and treated with penicillin or amoxicillin monotherapy. Of these children, 527 (30.8%) were enrolled in the clinical trial and 1,182 (69.2%) received routine care. Two-thirds of the children (1,242/1,709) studied were hospitalized at the four district hospitals. Study participants in both groups had a median age of 13 months [interquartile range (IQR): 7–24 months] and duration of symptoms of 3 days (IQR: 2–4 days). Sex, immunization status, duration of symptoms, nutritional status, and the presence of pallor were comparable between the two groups (Table 1). In contrast, history of a wheeze was reported three times more frequently among children enrolled in the clinical trial (35.4% vs. 11.2%; *P* < 0.01); dehydration was more common among children in the observational cohort (8.6%) than those in the clinical trial (5.9%), *P* = 0.05; and the proportion of children enrolled from district hospitals was greater among nontrial (75.0%) vs. trial (67.4%) participants (*P* < 0.01). More than 90% of the children for whom information on immunization status was available were reported to have received the routine childhood vaccines, including the pneumococcal conjugate vaccine, according to the national immunization schedule. Data on patient characteristics and outcome were documented completely for all the clinical trial participants. Among children in the observational cohort, data were missing on immunization status (10.0%), weight for age Z score (10.7%), history of a wheeze (45.0%), assessment for pallor (10.9%), and survival/death (3.6%).

**Table 1:** Baseline characteristics of study participants

Patient characteristic	Observational cohort		Clinical trial cohort		*P*-value
	Frequency (%)/median (IQR)	*N*	Frequency (%)/median (IQR)	*N*	
Age 12–59 mo	654 (55.3)	1,182	292 (55.4)	527	0.90
Age 2–11 mo	528 (44.7)		235 (44.6)		
Female	479 (41.0)	1,182	226 (42.9)	527	0.46
Male	703 (59.0)		301 (57.1)		
Immunization up to date	994 (93.4)	1,064	501 (95.1)	527	0.20
Immunization not up to date	70 (6.6)		26 (4.9)		
Duration of symptoms (days)	3 (2, 4)	1,182	3 (2, 4)	527	0.15
Weight-for-age Z score ≥2SD	917 (86.9)	1,055	447 (84.8)	527	0.25
Weight for Age Z score <−2SD^a^	138 (13.1)		80 (15.2)		
No history of wheeze	577 (88.8)	650	340 (64.6)	527	<0.01
History of wheeze	73 (11.2)		187 (35.4)		
Pallor absent	1,004 (95.3)	1,053	498 (94.5)	527	0.46
Pallor present	49 (4.7)		29 (5.5)		
Dehydration absent	1,080 (91.4)	1,182	496 (94.1)	527	0.05
Dehydration present	102 (8.6)		31 (5.9)		
District hospital^b^	887 (75.0)	1,182	355 (67.4)	527	<0.01
Provincial hospital	295 (25.0)		172 (32.6)		

a Weight for age Z score classification based on World Health Organization Child Growth Standards.

b Vs. provincial hospital.

### 3.1 Univariate associations for mortality

Mortality among nontrial participants for whom outcome data were complete was 14/1,140 (1.2%) vs. 4/527 (0.8%) for those enrolled in the trial [unadjusted odds ratio (OR), 0.6; 95% CI: 0.2–1.9]. Children aged 2–11 months were more than four times more likely to die than those aged 12–59 months (OR, 4.5; 95% CI: 1.5–13.7). More deaths were also observed among children who reported a duration of symptoms of 3 or more days compared to children with shorter durations of illness (OR, 4.0; 95% CI: 1.2–13.9) as well as those hospitalized at provincial-level facilities (OR, 2.7; 95% CI: 1.1–6.8). There was weak evidence that up-to-date immunization status was associated with reduced odds of death (OR, 0.3; 95% CI: 0.1–1.1). Mortality did not vary by sex, the presence of moderate malnutrition, history of a wheeze, nor the presence of pallor (Table 2).

**Table 2:** Univariate risk factors for mortality among study participants

Patient characteristic	Number of deaths/*N*	% Mortality	Odds ratio (95% CI)	*P*-value
Observational cohort	14/1,140	1.2	1.0	
Clinical trial cohort	4/527	0.8	0.6 (0.2, 1.9)	0.40
Age 12–59 mo	4/927	0.4	1.0	
Age 2–11 mo	14/740	1.9	4.5 (1.5, 13.7)	0.004
Female	8/692	1.2	1.0	
Male	10/964	1.0	0.9 (0.4, 2.3)	0.8
Immunization not up to date	3/92	3.3	1.0	
Immunization up to date	15/1,434	1.1	0.3 (0.1, 1.1)	0.06
Duration of symptoms < 3 days	10/1,174	0.4	1.0	
Duration of symptoms ≥3 days	8/493	1.6	4.0 (1.2, 13.9)	0.02
Weight-for-age Z score ≥2SD	11/1,334	0.8	1.0	
Weight-for-age Z score <−2SD	4/212	1.9	2.3 (0.7, 7.3)	0.14
No history of wheeze	11/899	1.2	1.0	
History of wheeze	1/259	0.4	0.3 (0.0, 2.4)	0.24
Pallor absent	14/1,471	1.0	1.0	
Pallor present	2/71	2.8	3.0 (0.7, 13.6)	0.13
Dehydration absent	16/1,537	1.0	1.0	
Dehydration present	2/130	1.5	1.5 (0.3, 6.5)	0.60
District hospital	9/1,184	0.8	1.0	
Provincial hospital	9/451	2.0	2.7 (1.1, 6.8)	0.03

*Abbreviation:* CI, confidence interval.

In bivariate analyses, the association between participation in the trial and mortality was modestly altered after adjusting for age group, malnutrition, history of a wheeze, pallor, and dehydration. There was no statistical interaction by any of the variables examined for the primary association (Table 3).

**Table 3:** Stratified and Mantel-Haenszel adjusted odds ratios for the association between trial enrolment and mortality

Patient characteristic	Stratum specific odds ratio (95% CI)	M-H summary odds ratio (95% CI)	*P*-value chi-square test	*P*-value test for interaction
Observational cohort	14/1,140		0.40	
Clinical trial cohort	4/527			
Age 12–59 mo	0.0 (–)	0.7 (0.2, 2.0)	0.46	0.22
Age 2–11 mo	0.9 (0.3, 3.0)			
Female	1.2 (0.3, 5.2)	0.6 (0.2, 1.9)	0.38	0.18
Male	0.2 (0.0, 1.9)			
Immunization not up to date	0.0 (–)	0.6 (0.2, 1.9)	0.38	0.37
Immunization up to date	0.7 (0.2, 2.3)			
Duration of symptoms < 3 days	0.0 (–)	0.6 (0.2, 1.9)	0.38	0.37
Duration of symptoms ≥3 days	0.7 (0.2, 2.3)			
Weight-for-age Z score ≥2SD	0.5 (0.1, 2.2)	0.7 (0.2, 2.3)	0.58	0.31
Weight-for-age Z score <−2SD	1.7 (0.2, 12.3)			
No history of wheeze	0.6 (0.2, 2.5)	0.8 (0.2, 2.7)	0.66	0.44
History of wheeze	0.0 (–)			
Pallor absent	0.8 (0.3, 2.7)	0.7 (0.2, 2.1)	0.48	0.32
Pallor present	0.0 (–)			
Dehydration absent	0.5 (0.1, 1.8)	0.7 (0.2, 2.0)	0.47	0.20
Dehydration present	3.3 (0.2, 54.9)			
District hospital	1.3 (0.3, 5.1)	0.6 (0.2, 1.8)	0.38	0.15
Provincial hospital	0.2 (0.0, 1.8)			

*Abbreviation:* CI, confidence interval.

### 3.2 Multivariate associations for mortality

Associations for mortality among trial vs. nontrial patients were further examined in a multiple imputation model adjusting for age group, hospital level of care, malnutrition, history of a wheeze, pallor, and dehydration. The result was similar to that obtained in the univariate analyses (adjusted OR, 0.7; 95% CI: 0.2–2.1). Over the whole sample (trial and observational cohort), children aged 2–11 months had a fourfold increased odds of death compared to those aged 12–59 months. Duration of symptoms and hospital level of care were, however, not independently associated with mortality (Table 4).

**Table 4:** Independent risk factors for mortality among study participants

Patient characteristic	Adjusted odds ratio^a^ (95% CI)	*P*-value
Observational cohort	1.0	
Clinical trial cohort	0.7 (0.2, 2.1)	0.51
Age 12–59 mo	1.0	
Age 2–11 mo	4.0 (1.3, 12.5)	0.02
Weight-for-age Z score ≥2SD	1.0	
Weight-for-age Z score <−2SD	2.0 (0.6, 6.7)	0.26
Pallor absent	1.0	
Pallor present	3.3 (0.6, 16.8)	0.15
No history of wheeze	1.0	
History of wheeze	0.4 (0.1, 2.7)	0.33
Dehydration absent	1.0	
Dehydration present	1.1 (0.2, 4.8)	0.93
District hospital	1.0	
Provincial hospital	2.2 (0.8, 5.6)	0.11

*Abbreviation:* CI, confidence interval.

a Multivariate model adjusted for trial versus no—trial status. age, weight for age Z score, pallor, history of wheeze, clinical hydration status, and level of care of health facility.

## 4 Discussion

In this comparative study of children hospitalized with pneumonia enrolled in a clinical trial vs. those who received routine care, overall mortality was low and similar in both groups. Our findings are consistent with the results of previously conducted clinical trials that informed a major recent revision in the WHO guidelines for the management of childhood pneumonia recommending outpatient therapy for children with lower chest wall indrawing with oral amoxicillin. The low mortality, however, contrasts with the results of previous observational studies conducted in sub-Saharan Africa prior to the introduction of the pneumococcal vaccine in which mortality was more than three times higher [15], [16]. This difference may be partly attributed to the wide immunization coverage against the major causes of bacterial pneumonia, resulting in a relative increase in the proportion of children presenting with self-limiting viral pneumonia [17]. Unfortunately, the etiology of pneumonia in low-income settings in the era postintroduction of the *Haemophilus influenzae* type B and pneumococcal conjugate vaccines remains poorly understood [17], [18] and was not examined in our study.

Most baseline characteristics were evenly distributed between the two groups. The disproportionately larger number of children in the trial with history of a wheeze, a symptom associated with viral bronchiolitis and reduced risk of death among young children with pneumonia [19], may suggest biased recruitment of patients who were perceived to be less sick in this group. However, the apparent difference may also be due to inadequate assessment for the presence of this symptom among nontrial participants (documentation on history of a wheeze was missing for almost half of nontrial participants). To mitigate measurement error arising from missing data, we used multiple imputation to generate the final estimates of effect. Since data for the children included in the observational cohort were collected retrospectively, it was not possible to ascertain the characteristics of this subpopulation in detail. Thus, the presence of underlying malnutrition, HIV infection, and other comorbidities that were unrecognized or undocumented by the admitted clinician could not be accounted for among the nontrial participants. While these factors suggest dissimilarity between the two groups, the comparison conducted in this study provides an important picture of outcomes among these children in real life—a more relevant consideration for policymakers interested in understanding the risk of death within this population.

Out of the large number of published pragmatic trials, very few attempts to demonstrate the extent to which the populations studied compare to real patients to whom the findings ultimately apply. Indeed, trials designed with the intention of generating evidence to be used in “real-world” settings have been criticized for being more explanatory than initially intended, limiting their utility for decision-making [20]. The emergence of tools to aid trialists in designing studies that appropriately match their intended purposes is likely to improve the quality of both explanatory and pragmatic trials [21], [22]. The possibility of selection bias can never be completely excluded in a study comparing RCT with observational evidence as pointed out above. Certainly, if results differ, the possibility that one group had worse prognosis than the other cannot be excluded. Nor, if they are similar across the trial and nontrial comparators, as in this case, is it completely safe to assume that a true difference has not been obscured. That said, the similarity in both the absolute and relative differences across trial and nontrial groups, in both the adjusted and unadjusted results, does suggest that a measure of reassurance might be derived that the trial result is generalizable in this particular case.

A major limitation of this study was its retrospective design and reliance on routine clinical data. Missing data were encountered across several variables including clinical outcome. Although it is not possible to ascertain the true values of the missing observations, we used multiple imputation to overcome potential bias arising from exclusion of patients with missing data. The results of this analysis were similar to those of the univariate analysis excluding children with missing outcome data.

## 5 Conclusions

Routinely collected observational data, when used to complement the findings of clinical trials, offer a valuable and low-cost opportunity for addressing the weaknesses inherent to traditional RCTs [23], [24]. The validity of this approach is, however, dependent on the completeness and quality of clinical documentation which is currently a major challenge in settings where resources are deficient [25], [26]. Nontrial participants had similar characteristics to those recruited in the RCT. The risk of mortality in routine care settings was comparable to that reported in clinical trials, among children who are currently proposed to receive outpatient therapy under the revised WHO guidelines. The findings add to existing evidence to further inform ongoing discussions on case management for childhood pneumonia in sub-Saharan Africa.
